# Health equity follows racial equity: learning the impact of historic racism through a summer reading assignment in a graduate public health course

**DOI:** 10.3389/fpubh.2025.1601195

**Published:** 2025-06-25

**Authors:** Donna J. Petersen

**Affiliations:** College of Public Health, USF Health, Tampa, FL, United States

**Keywords:** pedagogy, public health, graduate education, historic racism, health equity

## Abstract

Prior to a fall semester graduate course in public health, students are encouraged to complete a summer reading assignment based on a book chosen from a curated list of popular literature relevant to public health concepts. From 2020 to 2024, this assignment enabled students to explore the history of structural and systemic racism in the United States and its impacts on current public health policy and programs. Themes from these books are woven into class discussions and further explored in subsequent course assignments. This paper describes the assignment and shares responses from the 1,141 students who completed the reading assignment as well as responses to subsequent assignments completed by the 1,464 students enrolled in the course. Scaffolding of complex and for some students challenging subjects facilitates a deeper understanding of factors critical to understanding contemporary public health challenges and helps students appreciate the significance of the social economic, and political determinants of health, including racism.

## Introduction and background

Public health as a societal endeavor is vast and complex. The governmental workforce in every state, charged with the responsibility for promoting and protecting the public’s health, is multidisciplinary, engaged in a comprehensive array of programs, policies, and activities that assure health through a variety of mechanisms, including partnering with myriad agencies and organizations that contribute to a public health system. Creating the conditions that allow everyone in a population to be healthy is no small task and requires active engagement across a community. Appreciating the diversity of communities and the disproportionate impacts of systems and structures on their health is essential to understanding the root causes of disparities, building trust, and achieving the goal of health equity. Preparing professionals to work in this environment is a complex undertaking.

Schools and programs of public health accredited by the Council on Education for Public Health (CEPH) follow a set of standards in curriculum design and delivery (1). Recognition of the need to directly address the experiences of racial and ethnic minorities in approaching public health goals in public health curricula evolved slowly. Indeed, the first official acknowledgement that racial disparities in health existed in the United States was the 1985 USDHHS Secretary’s Report on Black and Minority Health (2). As early as 1980, CEPH criteria mentioned affirmative action in student admissions and the need to assure that “stated application, admission, and degree-granting requirements [are] applied equitably.” In 1993, CEPH revised the criteria to include the requirement that a school “recruit, retain and promote a diverse faculty,” and despite the Institute of Medicine’s 2003 recommendation that “cultural competence” be included in graduate education programs (3), “Diversity” did not become its own accreditation criteria category until 2011. These criteria included requirements that schools demonstrate “a commitment to diversity” and “cultural competence in learning, research and service practices (4).”

Before extensive revisions were introduced in 2016, CEPH required a core curriculum for the MPH based on what at that time were considered the five core disciplines of public health (i.e., biostatistics, environmental health sciences, epidemiology, health policy and management, and social and behavioral sciences). These five courses, each devoted to a specific skill set or content area, were intended to provide depth of knowledge in areas relevant to public health practice, but by design were taught separately, precluding the exploration of foundational and shared concepts. The first effort by the Association of Schools of Public Health to articulate a set of competencies for MPH graduates addressed this by adding seven cross-cutting competency areas, one of which was diversity and culture (5).

It is imperative to assure students of public health develop competence in definitions, foundational principles, structures and functions, and values of public health. This theme was repeated throughout the work of the Framing the Future Task Force, launched by ASPH in 2011 and concluded in 2015 (6). These conversations informed the most significant revision of CEPH standards since its founding in 1974 most notably a decided shift away from a siloed model of teaching elements of public health toward greater flexibility in assuring students developed foundational knowledge and achieved foundational competence. Within the new domain of foundational knowledge was included “explain public health history, philosophy and values” (D.1.1.), “list major causes and trends of morbidity and mortality in the U. S. or other community relevant to the school or program, with attention to disparities among populations, e.g., socioeconomic, ethnic, gender, racial, etc.” (D.1.4.), and “explain the cultural, social, political and economic determinants of health and how the determinants related to population health and health inequities” (D.1.10) (7). Within the domain of foundational competencies was included “discuss the means by which structural bias, social inequities and racism undermine health and create challenges to achieving health equity at organizational, community and systemic levels” (D.2.6.) (7). This new language made clear the importance of addressing issues of diversity, equity, and historic racism in public health curriculum and encouraged innovation in how this could be accomplished (7).

## Pedagogical approach

The College of Public Health at the University of South Florida took early and full advantage of this changing landscape, transforming its required core curriculum for the MPH degree from five separate courses into an integrated, competency-focused curriculum, team-taught by faculty from across the College (8–10). First offered in 2014, the transformed core curriculum begins with a 1-credit course taught over the first 5 weeks of the fall semester titled “History and Systems of Public Health.” This course is designed to introduce to all new graduate students (including students in MPH, MSPH and MHA programs and any PhD and DrPH students who have not previously completed a core curriculum) fundamental concepts, definitions, values, and the historical underpinnings of contemporary practice in public health. The first offering of the course was heavily evaluated and in response to student feedback and faculty experience, a summer reading assignment was added to the syllabus for this course.

From 2015 to 2019, the curated summer reading list offered students a choice of books, each addressing an element of history in the evolution of public health (see Table 1 for the original curated list). Because of the persistent impact of the history of racism in the United States on public health, the list always contained at least one book on this subject. Following the murder of George Floyd in 2020, the list was revised to include only books on the history of structural and systemic racism in the United States (see Table 2 for a list of the books that have been on the curated list at least once since 2020, and the years they appeared on the list). The assignment has always been voluntary; satisfactorily completing it adds five points to the final exam score. Most students choose to complete the assignment which is presented to them as follows:

**Table 1:** Books included in the original summer reading assignment.

Gawande, Atul. 2014	Being Mortal: Medicine and What Matters in the End, Metropolitan Books.
Johnson, Steven. 2006	Ghost Map: The Story of London’s Most Terrifying Epidemic and How it Changed Science, Cities and the Modern World, Riverhead Books.
May, Elaine Tyler. 2010	America and the Pill: A History of Promise, Peril and Liberation, Basic Books.
Quinones, Sam. 2015	Dreamland: The True Tale of America’s Opiate Epidemic, Bloomsbury Press.
Richtel, Matt. 2015	A Deadly Wandering: A Tale of Tragedy and Redemption in the Age of Attention, Mariner Books.
Salsburg, David. 2002	The Lady Tasting Tea: How Statistics Revolutionized Science in the Twentieth Century, Holt Paperbacks.
Wilkerson, Isabel. 2010	The Warmth of Other Suns: The Epic Story of America’s Great Migration, Vintage Books.
Wise, Tim. 2015	Under the Affluence: Shaming the Poor, Praising the Rich and Sacrificing the Future of America, City Lights Open Media.

PHC 6488: history and systems of Public Health, 2015–2019, USF College of Public Health.

**Table 2:** Complete list of books ever included in the summer reading assignment (years included).

Author and Year	Title and (Years) Included in the List
Anderson, Carol. 2017	White Rage: The Unspoken Truth of Our Racial Divide, Bloomsbury Press. (2020)
Coates, Ta-Nehisi. 2015	Between the World and Me, One World Books. (2020–2021)
DiAngelo, Robin. 2018	White Fragility: Why it’s so Hard to Talk to White People about Racism, Beacon Press. (2020–2021)
Eberhardt, Jennifer. 2020	Biased: Uncovering the Hidden Prejudice That Shapes What We See, Think, and Do, Penguin Books (2020–2022)
Geronimus, Arline T. 2023	Weathering: The Extraordinary Stress of Ordinary Life in an Unjust Society, Little, Brown Spark (2024)
Kendi, Ibram X. 2017	Stamped from the Beginning: The Definitive History of Racist Ideas in America, Bold Type Books (2020–2021)
Kendi, Ibram X. 2019	How to be an Antiracist, One World Publishers. (2020–2021)
Kendi, Ibram X. and Keisha N. Blain, editors. 2021	Four Hundred Souls: A Community History of African America 1,619–2019, One World Publishers, (2022–2024)
McGhee, Heather. 2021	The Sum of Us: How Racism Costs Everyone and How We Can Prosper Together, One World Publishers. (2022–2024)
Metzl, Jonathan. 2020	Dying of Whiteness, Basic Books. (2020–2021)
Oluo, Ijeoma. 2019	So, You Want to Talk About Race, Seal Press. (2020–2021)
Sandler, Les. 2021	The Centennial Scandal: The Election of 1876, available on amazon.com (2023–2024)
Wilkerson, Isabel. 2010	The Warmth of Other Suns: The Epic Story of America’s Great Migration, Vintage Books (2020–2024)
Wilkerson, Isabel. 2020	Caste: The Origins of our Discontents, Random House. (2022–2024)
Wise, Tim. 2015	Under the Affluence: Shaming the Poor, Praising the Rich, and Sacrificing the Future of America, City Lights Publishers. (2020)

PHC 6488: History and Systems of Public Health, 2020–2024, USF College of Public Health.

Choose one of the books from the attached list and read it in its entirety.

Prepare a no more than two-page response to the following questions:

1. Include your name and the title of the book you chose.
2. What about this book interested you? Why did you choose it?
3. Did the book help you learn about the history of racism in the United States? Please share one or two things you learned that you did not appreciate before reading this book.
4. What is your general understanding of what public health means?
5. Did this book provide you any insight into how racism may impact public health? Please provide at least one example.

Subsequent assignments including writing assignments and a question on the final exam, give students opportunities to further explore these issues and develop a deeper understanding of the persistent impacts of this history on health status in the United States and efforts to promote health equity and eliminate health disparities (see Table 3). Of the four writing assignments in the first 4 weeks of the five-week course, three give students the opportunity to further explore the impact of historic racism on public health. In week one, students are asked to develop their own definition of public health and “to write one to two paragraphs describing an idea you have for a change in a policy specific to a system or a structure that could begin to address racial equity toward promoting health equity.” The third writing assignment asks students to provide an epidemiologic assessment of an issue related to a specific topic in the health care system that may be contributing to persistent health disparities. The fourth and final writing assignment gives students an opportunity to write a Letter to the Editor on a topic of their choosing. Finally, every year on the final exam there is at least one question that invites further discourse on the impacts of historic racism on public health. These questions have asked students to consider adding a “social determinant of health” to the list of essential benefits required under the Affordable Care Act, or to identify something that may be amenable to change because it has become unacceptable to the larger society, or to discuss whether poverty leads to poor health or poor health to poverty. Each of these questions allows students to explore the impact of historic racism on current challenges in public health and to suggest opportunities to modify the resultant systems, structures, or policies toward greater improvements in health for entire populations.

**Table 3:** Progression of assignments in a fall semester public health graduate course.

Pre-course	Week one	Week three	Week four	Week six (one-week post-course)
Summer reading assignment	Writing assignment 1	Writing assignment 3	Writing assignment 4	Final exam short-answer question
Choose from a list of books on historic racism in the US, submit a reflection paper	Identify a policy impacting health equity	Analyze a health system issue contributing to health disparities	Write an Op Ed on an issue of your choice	Respond to a question on the impacts of historic racism on public health

## Results

Over the 5 years since the list was modified in 2020, a total of 1,141 students submitted the voluntary summer reading assignment out of 1,464 enrolled for a 78% completion rate. Students from other countries awaiting visas, or students awaiting financial aid decisions may not enroll in time to learn about and complete the assignment. Students who have completed the assignment with few exceptions submitted insightful, sometimes emotional, written responses to these questions. Particularly in response to question 3 (Did the book help you learn about the history of racism in the United States?) students say they found the readings “eye-opening” and the stories they told “powerful” and “humbling.” Others use words like “shocking,” “jarring,” “painful,” “shameful,” “disgusting” and “sad.” Some said they were “caught off guard” by what they learned, particularly by the persistence of racism in existing but also evolving systems and structures (e.g., school vouchers, or the school to prison pipeline). Many students note that they had limited exposure to this history, were unfamiliar with it, or felt misinformed about the history and its present-day manifestations. Students from other countries have more knowledge about this history but are shocked to learn that the vestiges of this history persist in shaping American policy and contributing to ongoing disparities. All agree in response to question 5 that racism impacts public health.

Responses to the first week’s writing assignment in which they are asked “to write one to two paragraphs describing an idea you have for a change in a policy specific to a system or a structure that could begin to address racial equity toward promoting health equity” tend to focus on the health care system, an area with which new students have more familiarity. Examples of the proposals they offer include increasing insurance coverage for more people, redistributing health care facilities to address disparities in access, increasing cultural competence and representation (e.g., creating “affirmative action” for applicants to health professions’ schools), expanding telehealth, and relieving medical debt. Proposals addressing elements of the system outside health care that have disproportionately negative impacts on the health of minority populations include traffic enforcement, food security, paid parental leave, fair housing, transportation, zoning, voting rights, and funding for public schools. Some unique responses include eliminating gerrymandering, increasing small business investments in low-income communities, expanding access to financial services including bank accounts and credit, and tighter controls on sub-prime and pay-day lenders. Students who completed the summer reading assignment tend to provide more comprehensive responses to this assignment.

Topics for the third writing assignment over the years have included infant mortality, maternal mortality, and climate change. Some examples of suggestions for ways the health care system could respond to differential impacts of climate change include new treatment protocols for children with asthma, establishing hydration centers during heat waves, supporting vector control measures to reduce tropical disease transmission, updating history and screening protocols to identify climate-related injuries or acute or chronic illnesses, advocating for stricter air pollution controls, “prescribing” and promoting greater access to healthy food, allowing clinic sites to serve as cooling centers during heat waves, expanding emergency response protocols to include extreme heat and drought, and expanding insurance to cover the entire population.

The instructions for the Letter to the Editor assignment give students wide latitude to address any issue they are passionate about; yet nearly all focus on issues important to health equity. Some, including maternal mortality, labor force diversity, mass incarceration, hate crimes, excessive policing, criminal justice reform, and racial discrimination directly address racial and health equity. But nearly every issue passionately expressed in these letters if addressed would reduce racial disparities and promote health equity. Examples include food insecurity, noise pollution, drug pricing, access to quality public education, homelessness, reproductive rights, clean water, banning assault weapons, human trafficking, sexually transmitted infections, safe infection sites, public transportation, school lunch programs, disinformation campaigns, paid sick leave for hourly workers, mental health, voting rights, and unemployment. As we near the end of the class, it is becoming evident to the students that public health must address the entire population if it is to succeed in its goals and as one of those overarching goals is health equity, attending specifically to the root causes of health disparities is essential if progress is to be made.

Focusing on the final exam question “identify one manifestation of systemic or structural racism that contributes to poor health outcomes that you believe is unacceptable,” in 2020, the first year of the assignment, the largest number, 42 of 274 students, addressed the unacceptably high rates of infant and maternal mortality among black woman, which was a topic we had discussed in class. Other issues identified by students that we had discussed in class included disparities in health care quality by race (29 students), differential access to health care or insurance by race (25 students) and food insecurity (12 students). Other issues identified that had not been discussed in class included redlining leading to housing segregation (37 students); inequitable funding of schools and poor quality of education (28 students); disproportionate rates of incarceration and police intervention by race (19 and 17 students respectively); income inequality (11 students); chronic stress contributing to a high allostatic load (9 students); lack of access to credit (5 students); bias in hiring and lack of upper mobility in the job market (4 students); and persistent disparities in life expectancy by race (3 students). Unique responses included gerrymandering, generational poverty, bias in standardized tests, voter suppression, and the digital divide.

Concerns among elected officials about ideological instruction in higher education in the State of Florida led to a change in the wording of this item on the final exam. In 2024, the question read “identify one social determinant of health that has a disproportionate negative impact on a vulnerable population in the United States that you believe is unacceptable.” The most frequent response (52 out of 245 students) was “race,” or “structural racism,” followed by income inequality and bias in hiring (37 students), unequal access to health care (34 students), an impoverished physical/built environment (33 students), food insecurity (23 students), poor quality education (21 students) and housing instability (20 students). All of these have their origins in historic racism. Less frequently mentioned topics with links to historic racism included inadequate transportation systems (5 students), bias in health care (4 students) and mass incarceration (2 students). Responses to the question without direct links to racism were provided by 17 out of 245 students and included anti-gender/trans/LGBTQ+ policies (9 students), immigration status (3 students), forced poverty for persons with disabilities (2 students), gun violence (2 students) and loneliness and social isolation (1 student).

## Discussion

It is not possible to say with certainty that the summer reading assignment, designed intentionally to expose students to a deeper understanding of historic racism and its vestiges influences what they consider and choose to write about later in the course. Scaffolding is a tool to help students consider increasingly complex issues. Choosing to introduce the issue of historic racism by assigning a popular book is intended to provide them with foundational knowledge in a more accessible format. It is also intended to assure them that they are not alone in contemplating these issues and to encourage a greater willingness to explore uncomfortable topics that are essential to public health. Reading can facilitate education; reading widely available popular books on difficult subjects can level set the class facilitating conversations, opening minds, and sparking discussions towards creative solutions to the enduring challenges of this history.

A review of the literature focused on education in the health professions revealed a wide array of approaches to teaching concepts related to health equity and racism with an apparent rise in interest in these topics following the murder of George Floyd in 2020. Programs employed a variety of teaching methods such as “just in time” learning (11), workshops (12, 13), intentional additions to curricula around historical and contemporary impacts of colonialism and slavery (14), and vertical immersion in a single issue, e.g., Black maternal mortality (15). One integrative review identified 55 published papers from a variety of disciplines and elucidated four strategies that were common to all: encounter, reflection, discussion, and activism (16). Encounter strategies include exposing faculty and students to content or experiences around racism: content examples included YouTube videos of historic events, commercials, podcasts, popular movies, and literature authored by persons of color (16). These encounters were then followed by reflection papers, guided discussions, and opportunities for students to co-learn or engage in community action (16).

Focusing solely on education in public health programs, a systematic review by Chandler et al. (17) identified 11 articles describing various methods to teach public health students about racial justice and health equity. Two of the eleven articles focused on racism or antiracism as a topic of interest, one examining historic racism as a structural determinant of health using a didactic lesson, workshops, and museum tours (18) and the other describing the development of an anti-racism competency applied across a public health curriculum (19). Only one paper was found describing the assignment of a popular book to facilitate student understanding of historic racism (20). Rosario et al. (20) had graduate students read “*The Color of Law: A Forgotten History of How Our Government Segregated America*” by Richard Rothstein, discuss case studies, interact with local leaders, and engage in experiential learning in the community.

The scant but growing body of literature on this topic reflecting myriad ways to approach discussions of historic racism suggests opportunities to explore and expand different models toward facilitating knowledge and skill acquisition in this important determinant of health. Schools and programs are encouraged to consider using a curriculum review model to examine their curricular offerings, such as that described by Perez et al. (21) and to publish their experiences with the pedagogical models they have employed to embed this content as a means of increasing our shared understanding. Collectively, academic public health should continue to discuss ways to include and improve this content, particularly in the wake of growing hostility to basic concepts of diversity, equity, and inclusion. We suggest a summer reading assignment is a simple, straightforward way to introduce difficult and complex topics toward richer discussions in the classroom and a deeper appreciation of the challenges and the opportunities of public health work. A summer reading assignment focused on historical systemic and structural racism signals to incoming cohorts of students that this issue is important to their future educational journeys and careers and frames public health as a complex, population-based, systems-driven field of endeavor. Choosing popular books rather than textbooks signifies that these topics are of interest to a much wider audience; several of these books have been on best seller lists. Over the years students have shared that they have read more than one of these books; that they have encouraged family members and friends to read one or more of these books; or that they read them together with a spouse or partner. Public Health is a profession that demands competence in a variety of skill sets, knowledge, and a set of shared values. It is also practiced in and with the public. The summer reading assignment brings at least part of that public to life and sets the stage for the complex educational and career journey to come.

## Data Availability

The data analyzed in this study is subject to the following licenses/restrictions: these “data” are derived from assignments submitted by students in a graduate course, protected under federal law. Requests to access these datasets should be directed to dpeters@usf.edu.

## References

[1] Council on Education for Public Health. (2024). Accreditation criteria, schools of public health and programs of public health amended. Silver Spring, MD. Available online at: https://media.ceph.org/documents/2024.Criteria.pdf (Accessed February 3, 2025).

[2] US Department of Health and Human Services. (1985). The report of the secretary’s task force on Black and Minority Health, Washington, D.C. Government Printing Office. Available online at: https://archive.org/details/reportofsecretar00usde/page/n3/mode/2up (Accessed February 23, 2025).

[3] Gebbie K Rosenstock L Hernandez LM. (2003). Who will keep the public healthy? Educating public health professionals for the 21st century. Washington, DC, National Academies Press. 25057636

[4] Council on Education for Public Health. Accreditation criteria for schools of public health. Washington, D.C.: Council on Education for Public Health (1980).

[5] Association of Schools of Public Health. Master’s degree in public health core competency development project version 2.3. Washington, D.C.: Association of Schools of Public Health (2006).

[6] Association of Schools of Public Health (2015). Framing the future, the second hundred years of education in public health. Washington, D.C. Available online at: https://aspph.org/our-work/initiatives/framing-the-future/ (Accessed February 7, 2025).

[7] Council on Education for Public Health. Accreditation criteria, schools of public health and programs of public health, amended 2016. Silver Spring, MD: Council on Education for Public Health (2016).

[8] Debate R Corvin J Wolfe-Quintero K Petersen DJ. Application of the intervention mapping framework to develop an integrated 21st century core curriculum. Part one: mobilizing the community to revise MPH core competencies. Front Public Health. (2017) 5:287. doi: 10.3389/fpubh.2017.00287 29164095 PMC5671995

[9] Corvin J Debate R Wolfe-Quintero K Petersen DJ. Application of the intervention mapping framework to develop an integrated 21st century core curriculum. Part two: translation of MPH core competencies into an integrated theory-based core curriculum. Front Public Health. (2017) 5:286. doi: 10.3389/fpubh.2017.00286 29164094 PMC5672008

[10] Corvin J Debate R Wolfe-Quintero K Petersen DJ. Application of the intervention mapping framework to develop an integrated 21st century core curriculum. Part three: curriculum implementation and evaluation. Front Public Health. (2017) 5:285. doi: 10.3389/fpubh.2017.00285 29164093 PMC5672009

[11] Ayyala MS Hill J Traba C Soto-Greene M Shaiu S DallaPiazza M. Teaching health equity in the time of COVID-19: a virtual look through the lens of structural racism. J Gen Intern Med. (2022) 37:2323–6. doi: 10.1007/s11606-022-07516-2 35710672 PMC9202964

[12] Legha RK Richards M Mabeza RM Gordon-Achebe K Kataoka S. Teaching the legacy of slavery in American medicine and psychiatry to medical students: feasibility, acceptability, opportunities for growth. MedEdPORTAL. (2023) 19:11349. doi: 10.15766/mep_2374-8265.11349 37766875 PMC10520221

[13] Simpson T Evans J Goepfert A Elopre L. Implementing a graduate medical education anti-racism workshop at an academic university in the southern USA. Med Educ Online. (2021) 27:1981803. doi: 10.1080/10872981.2021.1981803, PMID: 34813390 PMC8635611

[14] Banerjee AT Tan A Boston-Fisher N Dubois C-A LaFontaine A Cloos P . Embedding anti-racism in schools of public health: a pathway to accountability for progress towards equity. Can J Public Health. (2023) 114:872–7. doi: 10.17269/s41997-023-00796-z, PMID: 37410365 PMC10486309

[15] Pleasant V Kotian A Hammoud MM Maben-Feaster R. The importance of discussing the history of racism in medical student education. Clin Obstet Gynecol. (2024) 67:499–511. doi: 10.1097/GRF.0000000000000879, PMID: 39061123 PMC11272137

[16] Sumpter D Thurman W Wright M Johnson K Duplechain D Abbyad C. Art praxis: evidence-based strategies for antiracism teaching in nursing. Nurs Educ Perspect. (2023) 44:273–8. doi: 10.1097/01.NEP.0000000000001171 37594418

[17] Chandler CE Williams CR Turner MW Shanahan ME. Training public health students in racial justice and health equity: a systematic review. Public Health Rep. (2021) 137:375–85. doi: 10.1177/00333549211015665, PMID: 34011218 PMC8900229

[18] Dennis SN Gold RS Wen FK. Learner reactions to activities exploring racism as a social determinant of health. Fam Med. (2019) 51:41–7. doi: 10.22454/FamMed.2019.704337, PMID: 30633797

[19] Hagopian A West KM Ornelas IJ Hart AN Hagedorn J Spigner C. Adopting an anti-racism public health curriculum competency: the University of Washington experience. Public Health Rep. (2018) 133:507–13. doi: 10.1177/0033354918774791, PMID: 29847749 PMC6055294

[20] Rosario C Amin AA Parker C. [un]forgetting history: preparing public health professionals to address structural racism. J Public Health Manag Pract. (2022) 28:S74–81. doi: 10.1097/PHH.0000000000001432 34797265

[21] Perez J Leonard WR Neubauer LC. Developing equity-focused education in academic public health: a multiple-step model. Pedagogy Health Promot. (2021) 7:366–71. doi: 10.1177/23733799211045986

